# Dissecting the Chloroplast Proteome of the Potato (*Solanum Tuberosum* L.) and Its Comparison with the Tuber Amyloplast Proteome

**DOI:** 10.3390/plants11151915

**Published:** 2022-07-24

**Authors:** Shengxuan Liu, Tengfei Liu, Enshuang Wang, Yunxia Cheng, Tiantian Liu, Guogang Chen, Minrui Guo, Botao Song

**Affiliations:** 1 Key Laboratory of Horticultural Plant Biology, Ministry of Education, Huazhong Agricultural University, Wuhan 430070, China; liushengxuan1109@foxmail.com (S.L.); wangenshuang@126.com (E.W.); 13296517556@163.com (T.L.); songbotao@mail.hzau.edu.cn (B.S.); 2 School of Food Science and Technology, Shihezi University, Shihezi 832003, China; cgg611@163.com (G.C.); gmrshzu@163.com (M.G.); 3 Key Laboratory of Potato Biology and Biotechnology, Ministry of Agriculture and Rural Affairs, Huazhong Agricultural University, Wuhan 430070, China; 4 College of Horticulture and Forestry Science, Huazhong Agricultural University, Wuhan 430070, China; 5 College of Plant Science, Tarim University, Alar 843300, China; chengyunxia2018@163.com

**Keywords:** proteomics, chloroplast, amyloplast, photosynthesis, starch metabolism, *Solanum tuberosum*

## Abstract

The chloroplast, the energy organelle unique to plants and green algae, performs many functions, including photosynthesis and biosynthesis of metabolites. However, as the most critical tuber crop worldwide, the chloroplast proteome of potato (*Solanum tuberosum*) has not been explored. Here, we use Percoll density gradient centrifugation to isolate intact chloroplasts from leaves of potato cultivar E3 and establish a reference proteome map of potato chloroplast by bottom-up proteomics. A total of 1834 non-redundant proteins were identified in the chloroplast proteome, including 51 proteins encoded by the chloroplast genome. Extensive sequence-based localization prediction revealed over 62% of proteins to be chloroplast resident by at least one algorithm. Sixteen proteins were selected to evaluate the prediction result by transient fluorescence assay, which confirmed that 14 were distributed in distinct internal compartments of the chloroplast. In addition, we identified 136 phosphorylation sites in 61 proteins encoded by chloroplast proteome. Furthermore, we reconstruct the snapshots along starch metabolic pathways in the two different types of plastids by a comparative analysis between chloroplast and previously reported amyloplast proteomes. Altogether, our results establish a comprehensive proteome map with post-translationally modified sites of potato chloroplast, which would provide the theoretical principle for the research of the photosynthesis pathway and starch metabolism.

## 1. Introduction

The chloroplast, the energy organelle unique to plants and green algae, performs many functions. The primary role of the chloroplast is photosynthesis, which converts light energy to chemical energy. Additionally, the chloroplast plays a central role in amino acid metabolism, biosynthesis of fatty acids and various secondary metabolites [1]. Thus, the chloroplast is critical for plant productivity and survival. The chloroplast harbors an independent genome as a feature of cyanobacterial origin through endosymbiosis. The chloroplast genome contains a small set of genes encoding their photosynthetic machinery and various housekeeping functions, revealing that many genes were lost from plastids or transferred to the nucleus during evolution [2]. In many cases, proteins can be predicted at the cDNA or mRNA level by identifying deduced amino acid sequences. Therefore, global proteomic analysis facilitates the validation of such predicted proteins to a functional form.

These nuclear-encoded chloroplast proteins are synthesized in the cytoplasm and then transported to the chloroplast [3]. The chloroplast is bound by outer, inner, and thylakoid membranes, which compartmentalize the chloroplast into three soluble parts: the intermembrane space, stroma, and thylakoid lumen [4]. Delivery of proteins to the correct chloroplast internal compartments is involved in several targeting mechanisms. In addition to the proteins that target the outer envelope of chloroplast, most chloroplast proteins are imported into the stroma by an N-terminal targeting signal termed a transit peptide [5]. Nevertheless, some proteins without transit peptides are targeted to chloroplast via the secretory pathway [5]. Although chloroplast protein targeting was extensively studied, how chloroplast proteins are precisely delivered to distinct internal compartments remains to be elucidated entirely [6].

Clarifying the protein subcellular localization aids in characterizing the function of proteins. Proteomics is an effective way to explore cell organelles’ protein complements and gain new understandings of intracellular protein sorting and biochemical pathways. With the advancement of proteomics technology, many subcellular compartments in various plant species were illustrated by proteomic analysis [7,8,9,10]. The chloroplast proteome was widely investigated as a critical organelle in higher plants. For instance, in the model plant Arabidopsis, the chloroplast proteome was analyzed by MS along with various protein fractionation methods to assign proteins to chloroplast compartments [11,12]. Tamburino et al. [13] reported the tomato chloroplast proteome in response to drought stress and recovery. Wang et al. [14] illustrated the dynamics of chloroplast proteome in a salt-stressed mangrove (*Kandelia candel* L.). However, as the most critical tuber crop worldwide, the chloroplast proteome of potatoes has not been explored. Considering the chloroplast is also a crucial organelle for potato plants, characterization of the potato chloroplast proteome will facilitate deciphering the essential contribution of the chloroplast to potato plant growth and development.

Like the chloroplast, the amyloplast is bounded by a double membrane containing the stroma, where the starch granules are synthesized. All plastid-targeted proteins are thought to possess similar transit peptides and identical protein sorting systems [3]. However, there are essential differences between amyloplast and chloroplast; for instance, amyloplast is thought to be thylakoid-deficient. Since the amyloplast of potato tuber is rich in starch, its proteome attracts concern. Stensballe et al. [15] identified 27 and 20 proteins from mini- and micro-tubers amyloplast, respectively. Helle et al. [16] identified 36 proteins associated with potato tuber starch granules.

Moreover, there are profound differences in starch metabolism between potato chloroplast and amyloplast. In potato chloroplast, starch is synthesized as transient starch granules and degraded diurnally, supplying the energy needed for metabolism in the whole plant [17]. In potato amyloplast, starch accumulates as storage starch during tuber development, maintaining the energy requirements of the dormant tuber and fueling the outgrowth of new shoots after sprouting [17]. Comparative proteomics analysis to reveal different components between chloroplast and amyloplast has not yet been reported despite these differences. 

This study aims to analyze potato chloroplast proteome components and construct a high-quality reference proteome map for the potato chloroplast. In this study, we describe the proteome of potato chloroplasts. Several factors, including the well-established methods for isolating chloroplasts, the improved potato genome annotation released recently, and the sequenced potato chloroplast genome, greatly simplify the analysis of potato chloroplast proteome. Using high-mass-accuracy LC–MS/MS, we achieved a high proteome coverage of over 1800 proteins encoded by nuclear and chloroplast genomes and identified posttranslational modifications (PTMs). A comprehensive analysis of these proteins was conducted. We also evaluated the chloroplast proteome by transient fluorescence assay in tobacco epidermal cells. In addition, we analyzed the tuber amyloplast and compared similarities and differences between chloroplast and amyloplast proteome. Finally, we proposed starch metabolism pathways in potato’s chloroplast and amyloplast, revealing different regulatory mechanisms that operate in leaves and tubers. The chloroplast proteome and putative proteins identified will help build the groundwork for future research on the functional characterization of the chloroplast proteins in potatoes.

## 2. Results

### 2.1. Identification of Potato Chloroplastic Proteins

The chloroplast, a plant cell organelle of cyanobacterial origin, executes essential metabolic and biosynthetic functions. Due to the significant role of chloroplast in plants, it is necessary to discover the protein components in this subcellular organelle. To generate a reference proteome map of the potato chloroplast, we employed gel-free analyses followed by tandem mass spectrometry and searched against the improved potato protein database combined with the potato chloroplastic protein database to maximize the number of assignments. Using a false discovery rate (FDR) cutoff of 0.05 at the protein level, this approach resulted in 11,019 assigned non-redundant peptide groups representing 1834 protein groups, including 51 that are chloroplast genome-encoded (Supplementary Table S1). The average coverage was 19.0% (Supplementary Table S1).

### 2.2. Prediction Programs for Chloroplast Localization Recognize 62% of All Identified Proteins

**Figure 1:**
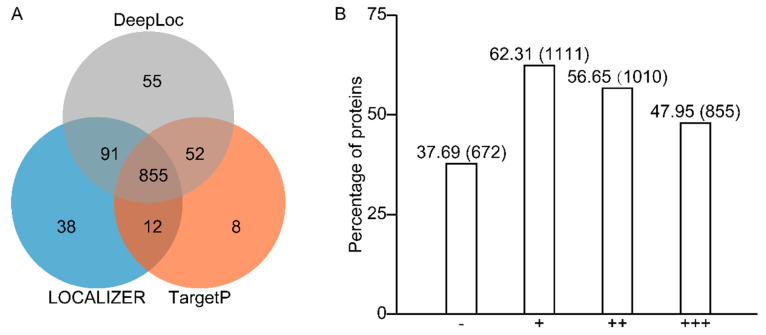
Chloroplast localization prediction for the identified 1783 nuclear-encoded proteins by TargetP, LOCALIZER, and DeepLoc. (**A**) The Venn diagram shows numbers of chloroplastic positives predicted by three different tools. (**B**) “+” “++” “+++” indicates prediction to be chloroplast by one to three programs, and “−” indicates prediction by none. Combination details of the predictions are given in Supplemental Table S2.

As the chloroplast-localized proteins may contain intrinsic transit peptides in their N-terminal region, chloroplast localization could be predicted using different programs to detect chloroplast transit peptides and cleavage sites. The identified 1783 nuclear-encoded proteins were subjected to localization prediction analysis with three different prediction programs: TargetP 2.0 (Department of Health Technology, Technical University of Denmark, Lyngby, Denmark), LOCALIZER, and DeepLoc 1.0 (Department of Health Technology, Technical University of Denmark, Lyngby, Denmark). As shown in Figure 1, each prediction tool returned a variable frequency of chloroplastic positives, ranging from 927 (TargetP) to 1053 (DeepLoc) of the potato proteomes. Of which 855 proteins were predicted to be chloroplast localized by all three programs, indicating consistent predicted results between programs. A total of 1111 out of 1783 proteins (62.31% of the total protein set) were predicted to be chloroplasts localized by at least one program, while 56.65% were predicted by two or more programs (Figure 1). Several are well-known chloroplast proteins among the 672 proteins that are not predicted to be localized by any programs. For instance, the starch metabolism-related proteins including protein targeting to starch 1 (PTST1, Soltu.DM.02G026830.4), phosphoglucomutase 1.1 (PGM1.1, Soltu.DM.03G016410.1), alpha-glucan phosphorylase 1a (PHO1a1, Soltu.DM.03G007760.1), putative phosphoglucomutase (pPGM, Soltu.DM.05G013630.1), triose-phosphate/phosphate translocator-like (TPT-like, Soltu.DM.01G008290.1), and isoamylase 3 (ISA3, Soltu.DM.06G000420.1 and Soltu.DM.06G000410.2), as well as photosynthesis-related proteins, were erroneously predicted to be localized out of the chloroplasts (Supplementary Table S2). Some proteins targeted to the chloroplast outer membrane are without any cleavable transient peptide, therefore, unable to be recognized by prediction tools.

A typical example Is outer envelope pore protein 34 (OEP34), located within the C-terminal membrane anchor’s 10-amino acid hydrophobic core [18]. This list of unrecognized proteins comprises two OEPs, OEP24A (Soltu.DM.04G035140.1) and OEP16-1 (Soltu.DM.06G004200.2) (Supplementary Table S2). The above results revealed that, except for proteins that are predicted to be chloroplastic, those which were not predicted to be such also included well-defined chloroplast proteins, suggesting that the proteins list obtained here provides a consolidated reference proteome map of potato chloroplasts.

### 2.3. The Distribution of Genes Encoded the Identified Proteins across the Chromosomes

**Figure 2:**
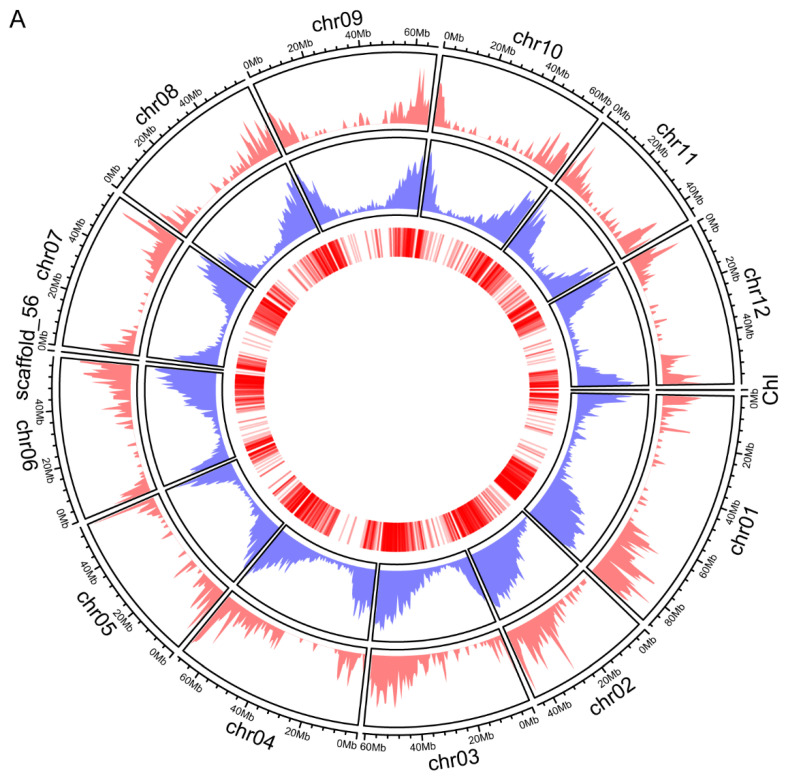

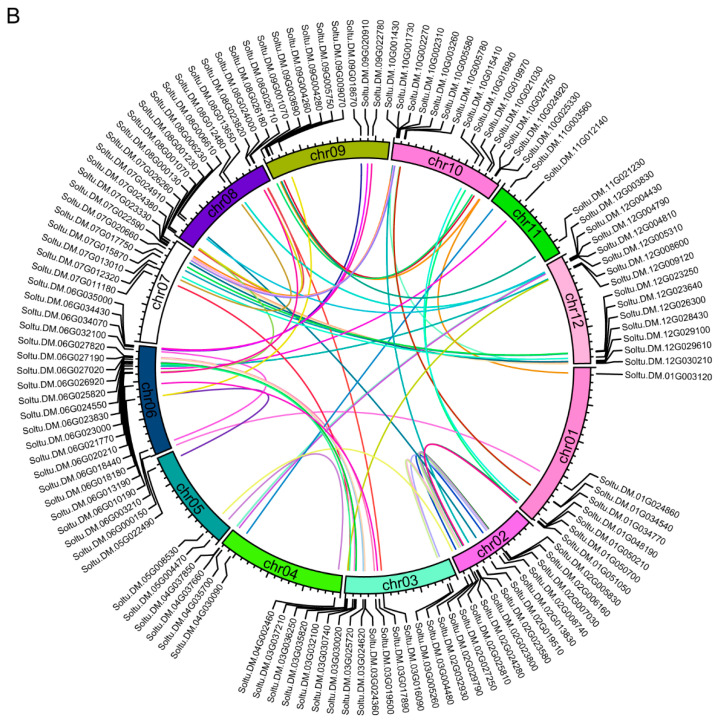
Chromosomal distribution of the identified proteins among nuclear and chloroplast genomes. (**A**) Circos plot represents 12 chromosomal DNA, a scaffold, and a chloroplast genome in a size-specific manner in its outermost peripheral ring. The pink layer and the blue layer indicate the chromosomal region and the number of proteins identified and all potato proteins, respectively, while the innermost circle represents the density of the identified proteins in each chromosome. (**B**) Circos plot shows the chromosomal distribution of duplicated genes encoded in these identified proteins.

Since the complete genome sequence of the potato is available, the distribution of genes that encoded the identified proteins across the chromosomes was analyzed. Chromosomal distribution and the gene density corresponding to the identified proteins were illustrated in a Circos plot (Figure 2A). Seventy-nine proteins are predicted to be chloroplast-encoded in the potato cultivar Desiree [19]. Our results provide experimental evidence of more than 64% of such proteins. Similar to the potato genome-wide gene distribution, these genes corresponding to the identified proteins exhibited no chromosome preference (Figure 2A). In addition, we identified many duplication events among these genes in potatoes using the MCScanX program (https://github.com/wyp1125/MCScanX, 21 October 2021). These duplication events involved 125 genes, implying that a portion of the genes encoding the chloroplast proteins were expanded through gene duplication (Figure 2B).

### 2.4. GO Analysis of the Identified Proteins

**Figure 3:**
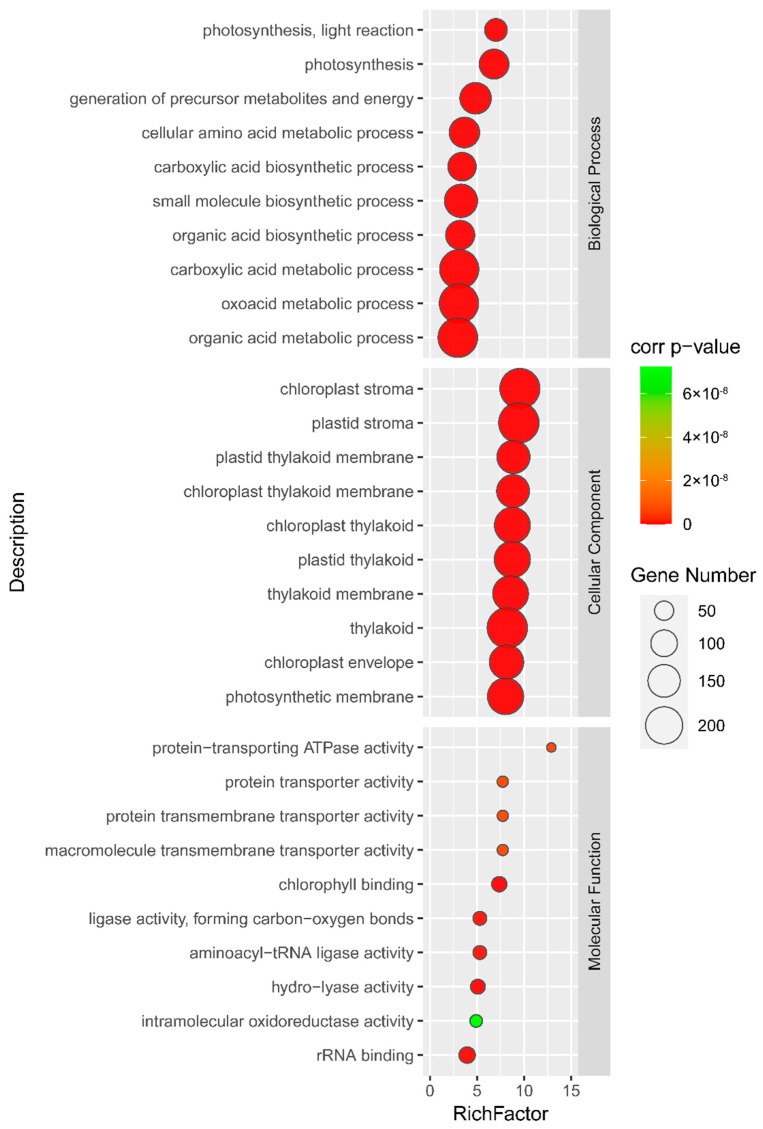
Statistics of GO enrichment analysis of the identified proteins. Gene ontology-based classification of the identified proteins according to their involvements in biological processes, cellular compartment, and molecular functions. The detailed information for the GO enrichment analysis is given in Supplementary Table S3.

To further characterize the function of potato chloroplast proteins, we analyzed the top 10 most significantly enriched gene ontology (GO) terms in respect of biological process, cellular component, and molecular function, respectively. As shown in Figure 3, as expected, the classification of the potato chloroplast proteins based on their unique biological process mainly included “photosynthesis”, “photosynthesis, light reaction”, “generation of precursor metabolites and energy”, and “cellular amino acid metabolic process”. Such proteins comprised photosystem I reaction center subunit N (Soltu.DM.08G005050.1), light-harvesting complex photosystem II subunit (Soltu.DM.01G044260.1), and phosphoglycerate kinase (Soltu.DM.07G028580.2) (Supplemental Table S3). Not surprisingly, for the cellular component, these chloroplast proteins had significant enrichment in “chloroplast stroma”, “plastid stroma”, and “plastid thylakoid” (Figure 3). Classification of the identified proteins according to their molecular functions revealed that “protein-transporting ATPase activity”, “protein transmembrane transporter activity” and “macromolecule transmembrane transporter activity” were the most enriching terms (Figure 3). GO analysis further demonstrated that these identified proteins mainly localized in chloroplasts and performed the functions of photosynthesis and generating metabolites. Such categorization will facilitate the characterization of the potato chloroplast proteins’ function and map them to specific pathways.

### 2.5. Evaluation of the Chloroplast Proteome by Transient Fluorescence Assay in Tobacco Epidermal Cells

**Figure 4:**
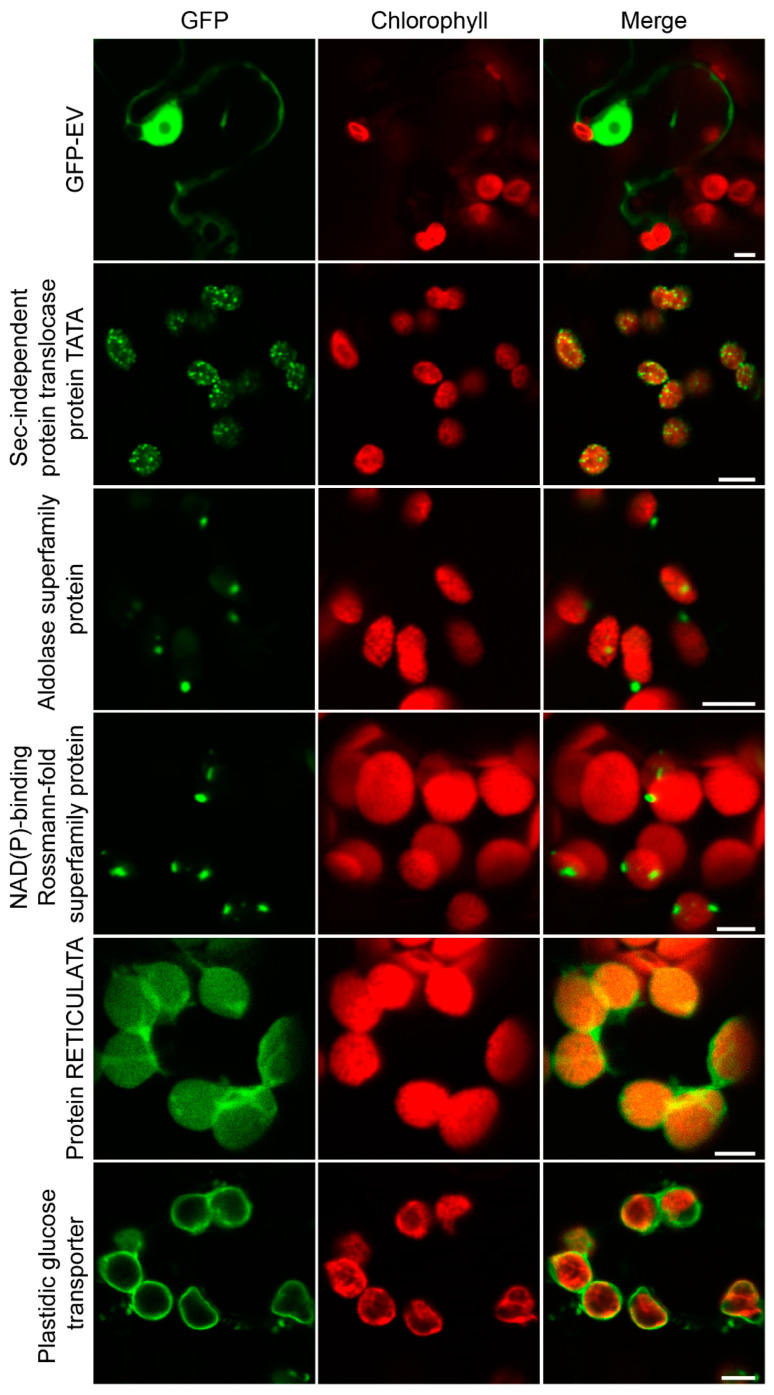
Subcellular localization analysis of representative identified proteins. The GFP alone and the GFP fusion constructs (genes-eGFP) were transiently expressed in N. benthamiana leaves via agroinfiltration. The images of epidermal cells were taken at 60 hpi by CLSM (TCS SP8 DLS, Leica, Wetzlar, Germany). 01G036490.1, Sec-independent protein translocase protein TATA; 05G004320.1, Aldolase superfamily protein; 06G027060.1, NAD(P)-binding Rossmann-fold superfamily protein; 09G001570.1, Protein RETICULATA; 02G026040.1, plastidic glucose transporter (pGlcT). Bars, 10 μm.

We randomly selected 16 proteins for further subcellular localization analysis to evaluate the chloroplast proteome data. An enhanced green fluorescent protein (eGFP) fluorescence tag was in-frame fused at their C termini to generate each gene-eGFP construct. The GFP fusion constructs (genes-eGFP) were transiently expressed in the model solanaceous plant *N. benthamiana* using *A. tumefaciens*-mediated expression with GFP empty vector alone (GFP-EV) as control. All 16 constructs could be detected in tobacco epidermal cells at variable levels, and 14 were validated to target chloroplasts (Figure 4, Supplemental Figure S1). Soltu.DM.09G004260.1- and Soltu.DM.01G049440.1-encoded putative histone deacetylase and cold, circadian rhythm, and RNA-binding proteins, respectively, were found to be localized to the nucleus, suggesting that these two were likely to be contaminants. The Soltu.DM.10G002680.1-encoded cobalt ion binding protein was predicted to be localized out of the chloroplast (Supplemental Table S2); however, it was demonstrated to target chloroplasts, suggesting the limitations of the current prediction tools. Although those 14 proteins were localized to the chloroplasts, the distribution patterns of the fluorescence signals of different constructs on the chloroplast are distinct. As shown in Figure 4, the GFP signals of Soltu.DM.01G036490.1 displayed dense granular evenly distributed on the chloroplasts, while that of Soltu.DM.05G004320.1 and Soltu.DM.06G027060.1 were observed to form one and two particles, respectively. Soltu.DM.02G026040.1 encoded a putative plastidic glucose transporter; therefore, it was not surprising that its GFP signals surround the chloroplasts. Soltu.DM.09G001570.1, which encodes a protein of unknown function, was evenly distributed on the chloroplasts, suggesting a stroma localization. The above observation revealed that those proteins are distributed in distinct internal compartments of the chloroplasts.

### 2.6. Posttranslational Modification Analysis of the Potato Chloroplast Proteins

**Figure 5:**
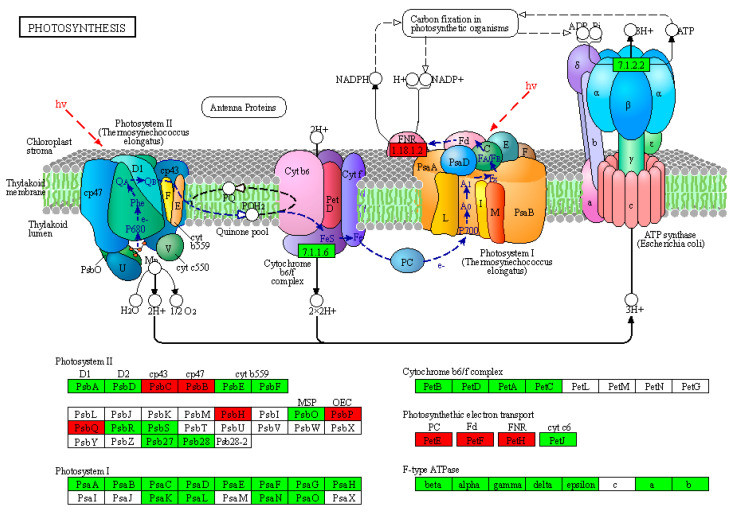
The KEGG photosynthesis pathway map for identified potato chloroplast proteins and phosphoproteins. The KEGG photosynthesis pathway map can be found online at http://www.kegg.jp/pathway/map00195 (21 October 2021). The green or red boxes indicate identified potato chloroplast proteins, while the red boxes represent the phosphoproteins. The detailed information on the KEGG photosynthesis pathway map is given in Supplementary Table S4.

Regarding numerous thylakoid proteins that are prominent amongst the phosphoproteins of plants, the potato chloroplast proteome was extensively searched for phosphorylation. We identified 136 phosphorylation sites in a total of 61 proteins. Individual proteins contained up to ten phosphorylation sites (Supplemental Table S1). KEGG analysis of these phosphoproteins found that 24 were involved in photosynthesis, including nine photosystem and electron transport system-related proteins and fifteen antenna proteins (Figure 5). Two paralogous protein kinases, STN7 (STATE TRANSITION7) and STN8 (STATE TRANSITION 8), which are essential for the phosphorylation of LHCII and the PSII core subunits, were counteracted by two protein phosphatases, PPH1/TAP38 (PROTEIN PHOSPHATASE1/THYLAKOID-ASSOCIATED PHOSPHATASE 38) and PBCP (PHOTOSYSTEM II CORE PHOSPHATASE) [20]. We searched against our chloroplast proteins and identified the corresponding homologous proteins comprising StSTN7 (Soltu.DM.12G018030.1), StSTN8 (Soltu.DM.10G021920.3), StTAP38 (Soltu.DM.03G022230.2), and StPBCP (Soltu.DM.06G002860.1) (Supplemental Table S1). Collectively, the above results implied that the regulation of thylakoid phosphorylation was conserved.

### 2.7. Comparative Proteomics between Chloroplast and Amyloplast

Since chloroplast and amyloplast have the exact developmental origin and share an identical genome, we aimed to perform comparative proteomics between chloroplast and amyloplast in the current study. In previous studies, by tryptic PMF of gel slices using conservative criteria for significantly identified proteins and combined with manual inspection of all MS data, 27 and 20 proteins were identified in mini- and micro-tubers, respectively [15]. In contrast, LC ESI-MS/MS analyses of the same tryptic digests and mascot analysis of data against an in-house potato protein database increased the number of significantly identified unique proteins to approximately 90 in both mini- and micro-tuber amyloplasts [15]. Proteome analysis of potato starch has identified a total of 36 proteins [16]. To facilitate the comparative analysis with our data, the peptides and proteins identified by the predecessors were searched against the improved potato protein database using sequence-based BLASTp analysis. Combined with all the non-redundant sets of potato amyloplast proteins, 204 proteins were obtained (Supplemental Table S5). Eighty-five proteins were common in chloroplasts and amyloplasts (Supplemental Table S5). This list of common proteins included 24 starch metabolism-related proteins (Supplemental Tables S5 and S7). While 1749 proteins were unique to the chloroplast proteome, 119 proteins were exclusively identified in the amyloplast proteome (Supplemental Table S5).

### 2.8. KEGG Pathway Illustrating Similarities and Differences between Chloroplast and Amyloplast Proteome

**Figure 6:**
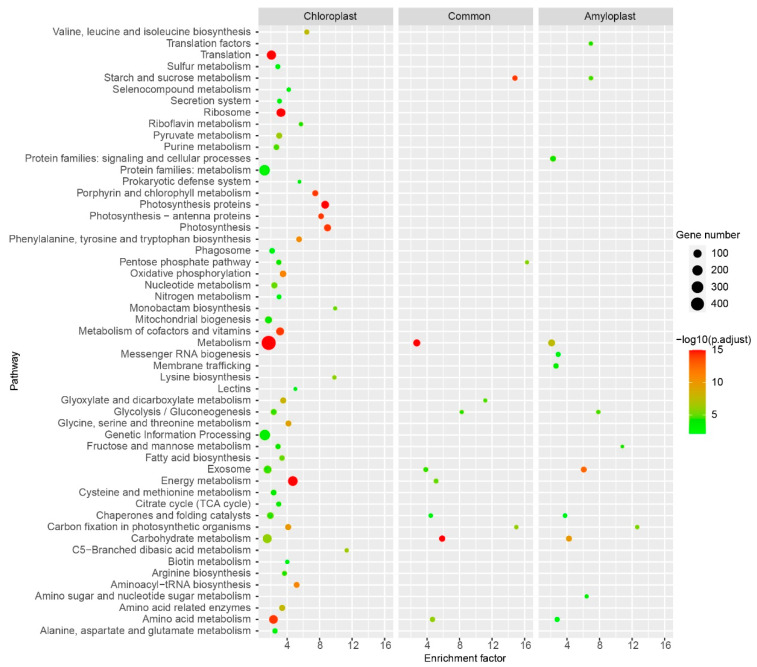
KEGG pathway analyses for chloroplast-unique protein set, amyloplast-unique protein set, and common protein set in chloroplasts and amyloplasts, respectively. The detailed information for these KEGG pathway analyses is given in Supplementary Table S6.

To further investigate the similarities and differences between the chloroplast and amyloplast proteome, we performed KEGG pathway analyses for chloroplast-unique protein set, amyloplast-unique protein set, and a common protein set in chloroplast and amyloplast, respectively. KEGG enrichment analyses identified 49 significant pathways, including seven pathways found in all three protein sets, five pathways found in two of three protein sets, five pathways found in the amyloplast-unique protein set, 36 pathways found in the chloroplast-unique protein set (Figure 6, Supplemental Table S6). For the common protein set in chloroplasts and amyloplasts, the most significantly enriched pathways included “Carbon fixation in photosynthetic organisms”, “Starch and sucrose metabolism”, and “Glyoxylate and dicarboxylate metabolism” (Figure 6), indicating the potato chloroplasts and amyloplasts shared many proteins in these pathways. The top three most significantly enriched amyloplast-unique protein sets were “Carbon fixation in photosynthetic organisms”, “Fructose and mannose metabolism”, and “Starch and sucrose metabolism” (Figure 6), revealing they were the central pathways in amyloplasts. Of the chloroplast-unique protein sets, the most significantly enriched pathways were “C5-Branched dibasic acid metabolism”, “Monobactam biosynthesis”, “Lysine biosynthesis”, and “Photosynthesis” (Figure 6). These chloroplast-specific pathways may be the characteristic features of the chloroplasts that distinguish them from amyloplasts. However, the enriched protein sets, including “Mitochondrial biogenesis”, “Citric acid cycle”, and “Exosome”, which are not chloroplast components, may be contaminations. These apparent contaminants contained 90 non-redundant proteins, accounting for approximately 4.907% of all identified proteins. The above results revealed that although both developed from the protoplasts, chloroplasts and amyloplasts displayed more differences than similarities.

### 2.9. Reconstruction of the Snapshots along Starch Metabolism Pathway in Chloroplasts and Amyloplasts

**Figure 7:**
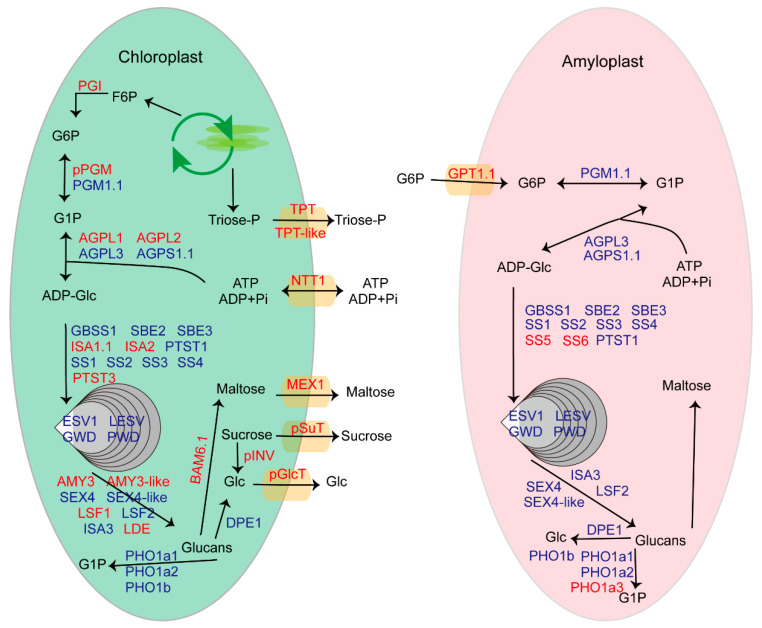
Proposed pathway of starch metabolism in different plastids. The proteins with red markers are unique in chloroplasts or amyloplasts, while these with blue markers are common in chloroplasts and amyloplasts. In the chloroplast, the Calvin–Benson cycle generates photoassimilates, including F6P and Triose-p. F6P can be converted to G1P in two subsequent steps catalyzed by PGI and PGM. G1P serves as the substrate for AGPase for starch biosynthesis. Triose-p can also be exported to the cytosol via the TPT. In the amyloplast, G6P synthesized via cytosolic PGM can be imported into the amyloplast by GPT. The imported G6P is then reconverted into G1P by plastidial PGM and as such can serve as a substrate for starch biosynthesis. F6P, Fructose-6-phosphate; Triose-p, triose-phosphate; TPT, triose-phosphate/phosphate translocator; PGI, phosphoglucoisomerase; G6P, glucose 6-phosphate; G1P, glucose 1-phosphate; PGM, phosphoglucomutase; AGPase, ADP-glucose pyrophosphorylase; SS, starch synthase; GBSS, granule-bound starch synthase; SBE, starch branching enzyme; GWD, glucan, water dikinase; PWD, phosphoglucan, water dikinase; BAM, beta-amylase; AMY, alpha-amylase; SEX4, starch excess 4; LSF, like starch-excess 4; DPE, disproportionating enzyme; PHO, alpha-glucan phosphorylase; GPT, glucose 6-phosphate/phosphate translocator; NTT, nucleotide translocator; pGlcT, plastidic glucose transporter; pSuT, plastidic sugar transporter; MEX, maltose transporter; ESV, early starvation; LESV, like ESV; PTST, protein targeting to starch; ISA, isoamylase-type starch debranching enzymes; SBE, starch branching enzyme; pINV, plastidic invertase. The protein accessions for the corresponding proteins are given in Supplementary Table S7.

The KEGG pathway analyses found that “Starch and sucrose metabolism” was one of the most significantly enriched pathways. In addition, considering the starch and sucrose metabolism are of considerable importance for potato biology, we aimed to reconstruct the snapshots along the starch metabolism pathway in the chloroplast and amyloplast at the protein level. We analyzed the starch metabolism-related proteins based on a previous genomic analysis of potato genes related to starch metabolism [17]. Except for the 77 loci identified by Van Harsselaar et al. [17], we added several recently reported proteins, for instance, plastidic sugar transporter (pSuT) [21], early starvation (ESV) [22], such as ESV (LESV) [22], and protein targeting to starch (PTST) [23]. After manual annotation, we identified 51 plastid-localized starch metabolism-related proteins, containing 24 proteins common in chloroplasts and amyloplasts, 19 proteins unique in the chloroplast, and four proteins unique in the amyloplast (Figure 7, Supplemental Table S7). The chloroplasts and amyloplasts shared many starch metabolism-related proteins, indicating similarities in their starch metabolism process. The chloroplast-localized starch metabolism-related proteins contained several sugar transporters, such as the maltose exporter MEX1 [24], the glucose exporter pGlcT [25], and the sucrose exporter pSuT [21], revealing these sugar exporters would play critical roles in the export of chloroplastic starch degradation products. The starch synthases SS5 and SS6 were explicitly identified in the amyloplasts. However, the expression of SS5 is detectable in potato leaves. Our chloroplast proteome was not recognized for SS5, possibly due to its low abundance. Additionally, the study in Arabidopsis has demonstrated that SS5 promotes starch granule formation despite low expression in leaves [26].

## 3. Discussion

The chloroplast executes essential metabolic and biosynthetic functions of vast significance, such as photosynthesis and amino acid biosynthesis [27]. Targeted proteomics have allowed high-throughput experiments on chloroplast samples, providing a comprehensive picture of the chloroplast proteome [28]. Understanding the components of the chloroplast proteome in the potato cultivar will give helpful information for potential yield. The present work aimed to unravel the total proteomic components of the potato chloroplast and generate a reference proteome map. By explicitly using the intact chloroplasts for performing proteomics analysis to diminish contamination, we successfully identified 1834 proteins with 5% FDR. We conducted a comprehensive in silico sequence analysis of the identified proteins to verify the dataset further. It was found that 51 proteins were encoded by the chloroplast genome, which accounts for more than 64% of the predicted potato chloroplast-encoded proteins (Figure 2). Over 62% of all identified proteins encoded by the nuclear genome were recognized by prediction programs for chloroplast localization (Figure 1 and Figure 2).

A comparison of three different subcellular localization prediction programs (TargetP, LOCALIZER, and DeepLoc) illustrated high numbers of overlapping positive predictions, indicating the convergence of the prediction methods applied by each tool. Generally, in at least one of the three prediction tools, 62% of the potato chloroplast proteome (not including chloroplast proteins) was predicted to localize in the chloroplast. Moreover, we selected 16 proteins by transient fluorescence assay in tobacco epidermal cells for further evaluation and found that 14 of 16 proteins were localized in the chloroplast (Figure 4, Supplemental Figure S1). Therefore, it can be concluded that our results provide experimental verification for the sequence-based prediction. However, authentic chloroplast proteins involved in the starch metabolism and the biosynthesis of amino acids and photosynthesis-related proteins were imprecisely predicted to be localized out of the chloroplast (Supplemental Table S2). These proteins might possess the noncanonical transit peptide for import into the chloroplast [29], which prediction tools would not recognize. Investigating the targeting mechanism of these chloroplast proteins that are not recognized by prediction tools would improve the accuracy of these programs.

The GO functional categorization of the proteins identified in our potato chloroplast proteome analysis reveals many photosynthetic complexes and metabolic and regulatory pathways (Figure 3). However, many components of metabolic pathways that were found in chloroplasts are not identified in our dataset. A rational explanation for this would be that the chloroplasts for proteomic analysis were under steady-state conditions; some chloroplast proteins may exist at specific developmental stages or under stress conditions (Rolland et al., 2012). Similar, in Arabidopsis, from an estimated ∼3000 proteins present in the chloroplast, chloroplast-targeted proteomics identified ranged from ∼600 to ∼1500 proteins [27,30,31,32,33].

Photosystem II (PSII), a dimeric complex, executes water-splitting at the onset of photosynthetic light reactions to fuel the electron transfer chain [34]. Five components of potato PSII were found to be phosphorylated (Figure 5), which is in line with previous reports on other species [35]. When we analyzed these phosphorylation sites in detail, we found that novel phosphorylation sites were also detected in addition to the conserved sites (Supplementary Table S1). For instance, Ser-391 in potato CP43 (ndhF), Thr-361 and Thr-365 in CP47 (PsbT), and Thr-90 in psbP (Soltu.DM.07G014630.1) were novel phosphorylation sites. Nevertheless, Thr-3 and Thr-5 in PsbH (rpl14) and Thr125 in psbQ (Soltu.DM.02G019510.1) were conserved phosphorylation sites [35] (Supplementary Table S1). A similar phenomenon for phosphorylation sites exists in the three photosynthetic electron transport-related proteins (Supplementary Table S1). Moreover, identifying critical protein kinases and phosphatases in our chloroplast proteome further revealed that dynamics of reversible protein phosphorylation occurred in potato thylakoids (Supplementary Table S1). It will be interesting to validate and characterize these novel phosphorylation sites in future work. The dynamic changes of these phosphorylation sites upon different environmental cues deserve in-depth investigation.

We conducted a comparative analysis between our chloroplast proteome and the previously reported amyloplast proteome. KEGG pathway analyses illustrated that the potato chloroplasts and amyloplasts displayed more differences than similarities (Figure 6). Moreover, we identified pathways including “C5-Branched dibasic acid metabolism”, “Monobactam biosynthesis”, “Lysine biosynthesis”, and “Photosynthesis” as the characteristic features of chloroplasts (Figure 6), which would help to uncover the complex metabolism pathways in the potato chloroplast. Additionally, the pathway of “Fructose and mannose metabolism” was enriched in both amyloplasts and chloroplasts; however, the pathway comprises distinct proteins in the two organelles (Figure 6), implying that the pathway has undergone functional differentiation in these two organelles.

In the present work, it was found that many starch metabolism-related proteins exist in both chloroplasts and amyloplasts (Figure 7), suggesting the conserved pathway of starch metabolism in these two organelles. Our results still revealed the difference between chloroplasts and amyloplasts. The plastidic sucrose transporter (pSuT) and plastidic invertase (pINV) were explicitly identified in chloroplasts, and their chloroplast localization was further verified by transient expression of GFP-tags (Supplemental Figure S2). Protein structure prediction results reveal that potato pSuT and pINV showed high protein structural similarity with corresponding functional homologs in Arabidopsis (Supplemental Figure S2). These results suggest that potato pSuT and pINV may be involved in chloroplast sucrose regulation.

Two isoforms of starch synthase, SS5 and SS6, were uniquely detected in amyloplasts. Due to the lack of the two catalytically active X-X-G-G-L motifs, the potato SS5 was considered an inactive isoform [16]. However, a recent study report that SS5 promotes starch granule formation in arabidopsis leaves. sIn contrast to SS5, SS6 is an activated isoform containing the catalytic amino acid residues of starch synthases [16]. Therefore, we hypothesize that SS5 and SS6 might play essential roles in the tuber storage starch formation. We still cannot rule out a function of SS5 in starch synthesis in potato leaves. Additionally, Glucose-6-phosphate (G6P) translocator (GPT) was performed as a plastid G6P/Pi antiporter that mediates the import of G6P into the amyloplast and export of Pi. In leaf chloroplasts, the ADP-glucose is generated from photo-assimilates within the Calvin–Benson cycle [36]. G6P is synthesized in the cytosol in the tuber and subsequently transported into the amyloplast, which is further metabolized to ADP-Glc [17]. G6P imported into amyloplasts is an essential process for starch biosynthesis in sink tubers. Hence, it was no surprise that GPT1.1 was uniquely identified in amyloplasts. In the present study, the amyloplast-specific accumulation protein SS5 was encoded by the corresponding tuber-specific expression gene according to the previous report [17]. This was also true for the chloroplast-specific accumulation protein AGPL1 [17]. These results support that protein levels are primarily determined by transcript concentrations on the bulk level and steady-state conditions [37].

Moreover, three sugar exporters were identified in the chloroplast. Future work to characterize their function in chloroplastic starch degradation product partitioning would be potentially significant for potato improvement. Starch metabolism-related proteins generally respond to environmental cues. Our data and those of our predecessors are only proteomes under certain conditions. The future isolation and identification of potato chloroplast and tuber amyloplast proteomes under different environmental cues will facilitate obtaining an exhaustive proteome.

## 4. Materials and Methods

### 4.1. Plant Material Preparation

The potato cultivar E3 was planted at Huazhong Agricultural University (Wuhan, Hubei Province, China; 30°28′ N, 114°21′ E). The plantlets of E3 were grown at the greenhouse (16 h Light/8 h Dark, 22 °C) from May to July 2021. Leaves without any damage, curling, wilting, yellowing, and disease were selected for the experiment.

### 4.2. Chloroplast Isolation

The isolation method of Percoll gradient centrifugation was based on the method of Kubis et al. [38] with some modifications. The plant materials were kept on the ice during the isolation procedure, and all the reagents were precooled at 4 °C. For the isolation of intact chloroplasts, 10 g of potato leaves were homogenized with 10 mL chloroplast isolation buffer (CIB, 0.3 M sorbitol, 5 mM MgCl_2_, 5 mM EGTA, 5 mM EDTA, 20 mM HEPES, 10 mM NaHCO_3_, 2 mM DTT, pH 8.0). The homogenate was filtered through two layers of Miracloth into a 50 mL centrifuge tube, and the residue was collected and homogenized with 10 mL CIB; repeated this step three times. The pooled, filtered homogenate was transferred into a 50 mL centrifuge tube and centrifuge at 1000 g for 5 min with the swinging-bucket rotor. The pellet was resuspended with 500 μL CIB, and the resuspended homogenate was transferred onto the top of the preformed Percoll gradient solution (100% Percoll solution was mixed with 2 × CIB in equal volume, 25 mL mixture was pre-centrifuged at 43,000× *g*, 4 °C for 30 min, stored at 4 °C until use). The gradient was centrifuged in a swing-out rotor at 6800 g for 20 min. After centrifugation, two bands were formed in the tube, and the intact chloroplasts were in the lower band. The intact chloroplasts were transferred to a precool centrifuge tube and added 20 mL wash buffer (3 M sorbitol, 50 mM HEPES, 3 mM MgSO_4_, pH 8.0). After inverting the tube softly to remove the Percoll gradient, the chloroplasts were centrifuged at 1000× *g*, 4 °C for 10 min. The pellet was resuspended by 500 μL wash buffer and collected as purified chloroplasts.

### 4.3. Protein Extraction and Preparation

Chloroplast proteins were extracted and digested by the previous study [39] with some modifications. All the steps were kept at 4 °C or on the ice during the extraction procedure. Chloroplast pellets obtained above were resuspended with a 10 × volume of TCA/acetone and precipitated overnight at −20 °C. The overnight precipitated sample was centrifuged at 12,000 rpm, 4 °C After discarding the supernatant, the pellet was washed with precooled acetone three times until the pellet became white or very light-colored. The washed pellet was dried in a fume hood overnight. The dried pellet containing chloroplast proteins was resuspended by an appropriate volume of lysis buffer (1% SDS, 100 mM Tris-HCl, 100 mM DTT, pH 8.0). The mixture was sonicated, followed by incubation for 30 min. Before starting sample processing, the lysate should be clarified by centrifugation at 12,000 rpm for 10 min. The supernatant was transferred to a 1.5mL microcentrifuge tube, and the protein concentration was mensurated by a Nanodrop. A total of 200 μg of chloroplast proteins for each sample were digested in a 10 kD filter using the FASP method as described [40]. After trypsin digestion, the samples were desalted and dried by vacuum. The peptides were reconstituted in mobile phase A (0.1% formic acid mobile).

### 4.4. MS Analysis

LC–MS/MS analysis was carried out using the Q Exactive Plus mass spectrometer (Thermo Fisher Scientific) coupled to the Easy nLC1200 nano-flow UPLC. Peptides (500 ng of each sample) were injected into the Acclaim PepMap 100 trap column (nanoViper C18, 100 μm × 2 cm, Thermo Scientific) and separated by the Acclaim Pep Map RSLC analytical column (nanoViper C18, 50 μm × 15 cm, Thermo Scientific) set at the flow rate of 300 nL/min. The solvent gradients were set as a linear gradient of 8~38% mobile phase B (80% acetonitrile contains 0.1% formic acid) over 102 min through 120 min run time. The sample was atomized using the nanoESI source. The data-dependent acquisition (Top 20) was carried out using MS survey scans in the 350~1700 m/z range with 70,000 mass resolution. For subsequent MS/MS analysis, the resolution was set to 17,500, and the isolation window was set to 1.6 m/z. The normalized collision energy was 27 eV. Peptides with charge 2~7 were selected.

### 4.5. Database Searching and Protein Identification

The mass spectrometry raw files were processed using Proteome Discoverer software version 2.4 (Thermo Fisher Scientific, Waltham, MA, USA). The potato nuclear-encoded protein database and the potato chloroplast-encoded protein database were downloaded from PGSC and NCBI, respectively. The MS/MS data were searched against the database using the SEQUEST algorithm. The search parameters included phosphorylation at threonine, serine, and lysine and N-terminal acetylation as variable modifications; 2 missed cleavages were allowed. The mass tolerance was set to 10 ppm for MS data and 0.05 Da for MS/MS. The proteins were identified at a 95% confidence level, and the false discovery rate was set to 5%. The mass spectrometry proteomics data were deposited to the ProteomeXchange Consortium via the PRIDE [41] partner repository with the dataset identifier PXD033047 and 10.6019/PXD033047

### 4.6. Bioinformatic Analysis

Localization prediction of the sequences were performed using three separate tolls: DeepLoc-1.0 (https://services.healthtech.dtu.dk/service.php?DeepLoc-1.0, 21 October 2021), TargetP-2.0 (https://services.healthtech.dtu.dk/service.php?TargetP-2.0, 21 October 2021), and LOCALIZER (http://localizer.csiro.au/, 21 October 2021). The distribution of the proteins across the chromosomal DNA of both nucleus and plastid was represented in the form of a Circos plot by the circlize package. GO enrichment analysis was carried out using the BiNGO plugin tool for Cytoscape 3.9.1 software (Institute for Systems Biology, Seattle, WA, USA) at the adjusted *p*-value < 0.01 with the Benjamini and Hochberg FDR. KEGG pathway analysis and Venn diagram analysis were conducted by TBtools v1.098736 software (South China Agricultural University, Guangzhou, China). AlphaFold2 through ColabAlphaFold2 (https://colab.research.google.com/github/sokrypton/ColabFold/blob/main/AlphaFold2.ipynb, 15 January 2022) project was used for protein structure prediction. Proteins structure visualization and analysis were conducted by UCSF ChimeraX (https://www.cgl.ucsf.edu/chimerax/, 15 January 2022).

### 4.7. Subcellular Localization Analysis

The coding sequences of the selected proteins (without the stop codon) were recombined into the pH7lic-C-GFP vector at the Stu I restriction site driven by the cauliflower mosaic virus 35S promoter. The recombined vectors were transformed into Agrobacterium tumefaciens GV3101 and infiltrated into tobacco (*Nicotiana benthamiana*) leaves [42]. For the colocalization assay, GFP and autofluorescence of chloroplast were captured by the laser scanning confocal microscopy (Leica TCS-SP8, Wetzlar, Germany) in multitrack line switch mode.

## 5. Conclusions

In summary, we established a reference proteome map of potato chloroplasts for the first time. Our potato chloroplast analysis identified 1834 non-redundant proteins, including 51 proteins encoded by the chloroplast genome. Among these nuclear-encoded proteins, over 62% of proteins could be recognized as chloroplast localization by at least one of the three prediction programs, revealing a relatively high purity of chloroplasts. Additionally, 14 out of the selected proteins are distributed in distinct internal compartments of the chloroplast, implying that the chloroplast proteome present here might encapsulate proteins with different subplastidial localization. The chloroplast also identified phosphoproteins and their putative kinases and phosphatases.

Furthermore, comparative analysis between chloroplast and previously reported amyloplast proteomes indicated that chloroplasts and amyloplasts displayed more differences than similarities. The starch metabolic pathways in the two different plastids were reconstructed based on the proteome data. Several sugar exporters or enzymes were found to be specific to the chloroplasts or amyloplasts. Future work to characterize their function will facilitate uncovering the difference between transient starch metabolism and storage starch metabolism. The result of this would be of potential significance for potato improvement.

## Data Availability

The data presented in this study are available on request from the corresponding author.

## Supplementary Materials

The following supporting information can be downloaded at: https://www.mdpi.com/article/10.3390/plants11151915/s1. Figure S1: Subcellular localization analysis of representative identified proteins. GFP fusion constructs (genes-eGFP) were transiently expressed in *N. benthamiana leaves* via agroinfiltration and the images of epidermal cells were taken at 60 hpi by CLSM. Bars, 10 μm; Figure S2: Subcellular localization and protein structure analysis of pSuT and pINV. (A) Subcellular localization analysis of pSuT and pINV. Bars, 10 μm. (B) Protein structure analysis of pSuT and pINV in potato and the arabidopsis. The protein structures are predicted by alphafold2. For two predicted structures of pSuTs from potato and arabidopsis, the root mean square deviation (RMSD) values between 438 pruned atom pairs is 0.410 angstroms (across all 537 pairs: 12.561); for two predicted structures of pINVs from potato and arabidopsis, the RMSD between 459 pruned atom pairs is 0.431 angstroms; (across all 617 pairs: 17.720); Table S1: The general information of the identified chloroplast proteins; Table S2: Combination details of chloroplast localization prediction for the identified 1783 nuclear-encoded proteins by TargetP, LOCALIZER, and DeepLoc; Table S3: The top 10 most significantly enriched gene ontology (GO) terms of the chloroplast proteins regarding the biological process, cellular component, and molecular function, respectively; Table S4: The detailed information of the KEGG photosynthesis pathway map for identified potato chloroplast proteins and phosphoproteins; Table S5: The proteins list for chloroplast-unique proteins set, amyloplast-unique proteins set, and common proteins set in chloroplast and amyloplast, respectively; Table S6: The detailed information of KEGG pathway analyses for chloroplast-unique proteins set, amyloplast-unique proteins set, and common proteins set in chloroplast and amyloplast, respectively; Table S7: The protein accessions information for reconstructing the proposed pathways of starch metabolism in different plastids.
