# Supplementary figures and images for: Dissecting the Chloroplast Proteome of the Potato (Solanum Tuberosum L.) and Its Comparison with the Tuber Amyloplast Proteome
Source: Plants (Basel). 2022 Jul 24;11(15):1915. doi: 10.3390/plants11151915 (PMC9332351; doi:10.3390/plants11151915)

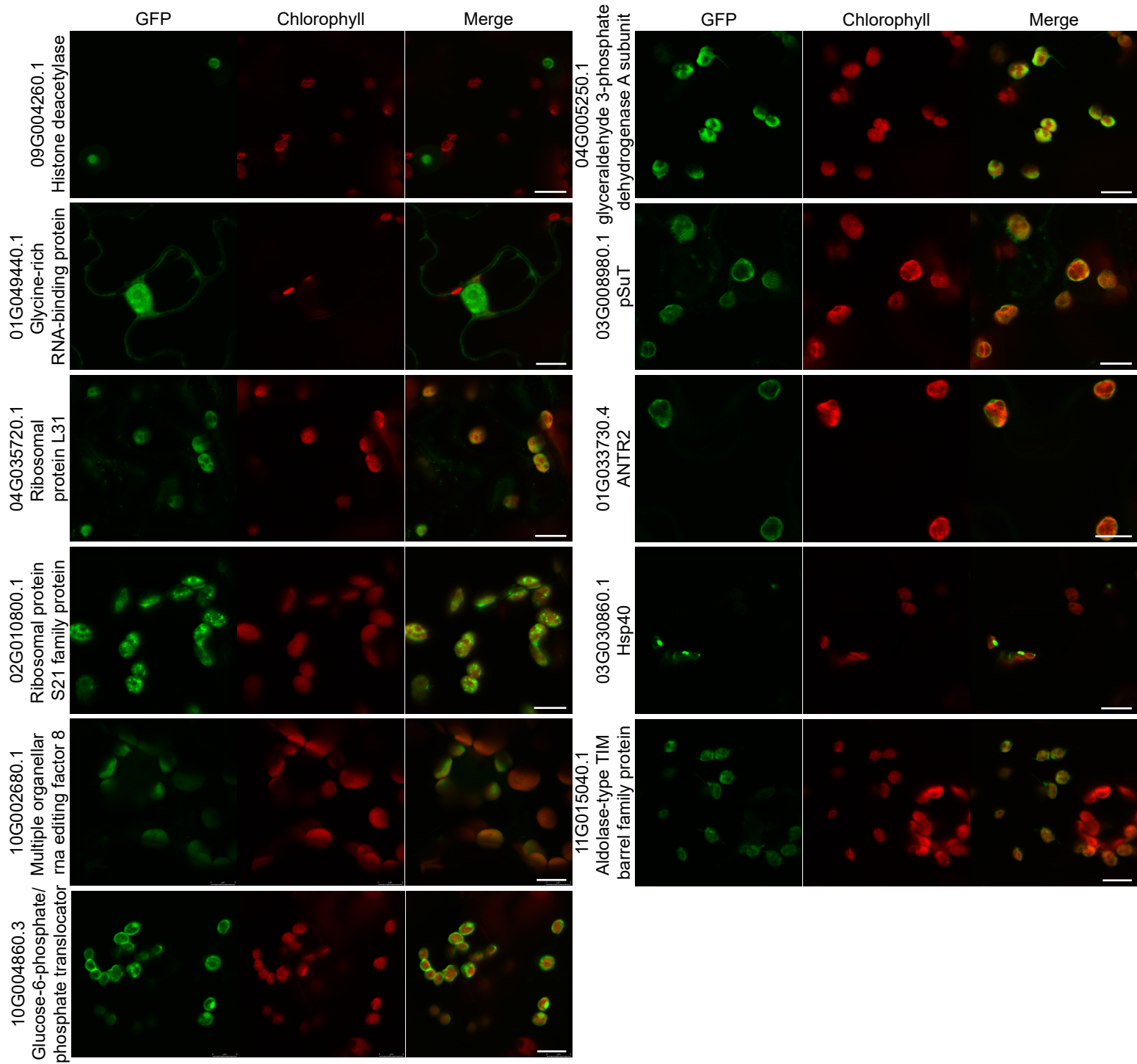

Supplement: Supplementary file 1 [file plants-11-01915-s001.zip › Figure S1.pdf]

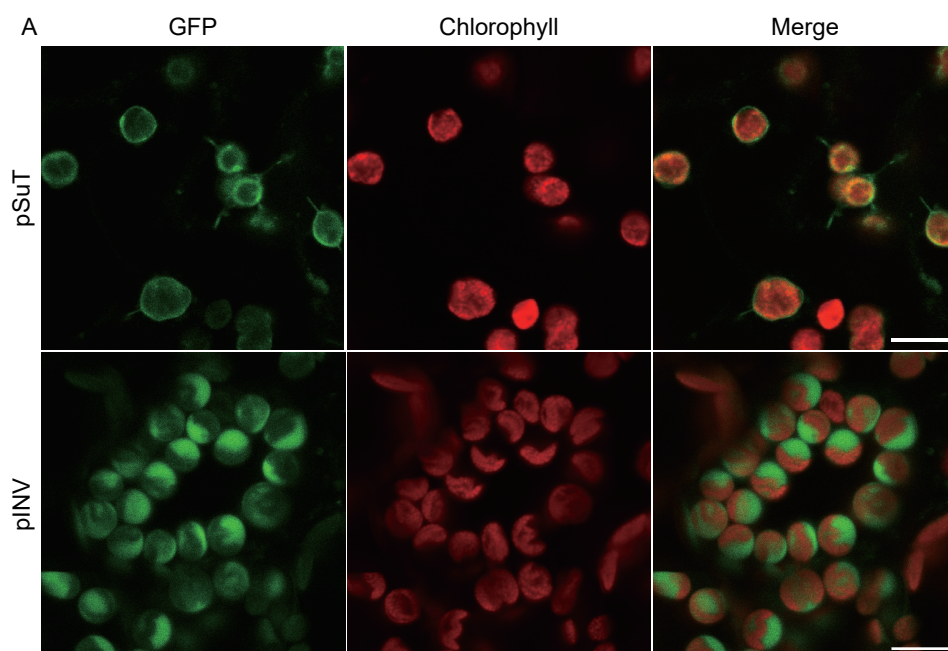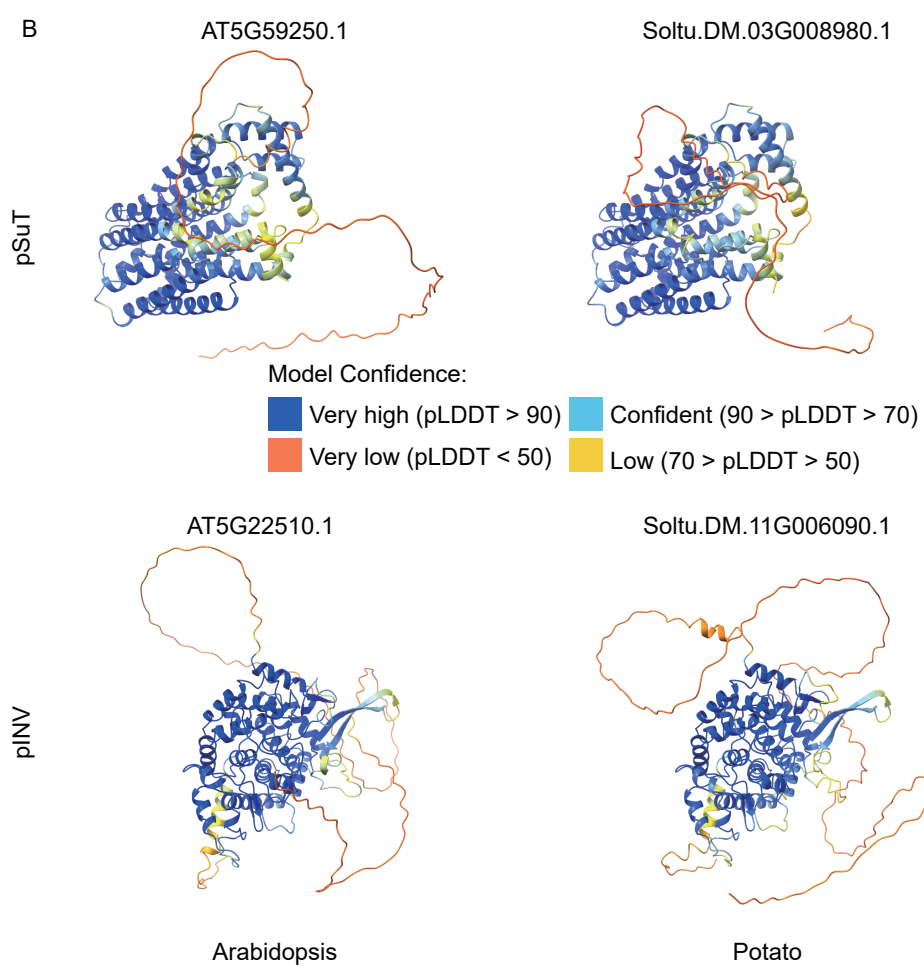

Supplement: Supplementary file 1 [file plants-11-01915-s001.zip › Figure S2.pdf]
